# Medical Items

**Published:** 1917-05

**Authors:** 


### MEDICAL ITEMS

The abstipation statis autotoxemia syndrome is complex in its aetiology as well as in its nosology. Anything that interferes with the calibre of the gut, or with the free passage of intestinal contents through the tube, results in a difficult passage of the bowel contents along the intestinal canal—Obstipation.

This may be a ptosis—or displacement of the gut at some point, a kink, abnormal sagging of suspensory structures, or dislocation of some part of the tube. This, together with abnormal dryness or lack of lubricating mucus, due to disturbance of the intestinal mucus glands, results in stagnation of the current, stoppage in many instances, a damming back of the current—Stasis.

As a result of these influences, opportunity is given for increased bacterial or chemical action, the production of an abnormal amount of toxins of unusual virulence, irritation and disturbance of the filtering or protective action of the mucous membrane and resulting absorption of increased quantities of poisonous material—Autotoxemia.

As a result of so many factors working more or less inter-dependently, is the establishment of the syndrome, a complex group of many symptoms, that may stimulate almost any disease or diseased condition met with in medicine.

Furthermore, these conditions, if allowed to go uncorrected, may and often do, aggravate and cause serious, even fatal disease.

The ideal treatment for such conditions is lubrication. The ideal intestinal lubricant is INTEROL because it comes close to Nature's own lubricant—mucuc—in that it lubricates without stimulation, irritation or enervation. Being non-absorable, it lubricates "all the way." On account of its characteristic **lubricating body,** it efficiently mixes, spreads and clings in the intestinal tract, and unless too much is administered it does not separate from the feces it lubricates and keeps soft. It does not "ooze." "per se."

---

### ECONOMY.

Although relatively unimportant, economy is a consideration by no means to be overlooked in the selection of a sedative for routine use. This has been especially true in the last eighteen months when the price of drugs of this class has been soaring to heretofore unheard of heights.

An examination of any complete drug price list reveals the fact that, with but one exception, no sedative can now be procured at as low a figure as before the war. The single exception is Neurosine· It is true that the price of this preparation was for almost a year 50 per cent. above the pre-war quotation, but on November 1, 1916, the price was reduced. Neurosine can now be procured at the old rate—8 oz. bottle, $1.00; 4 oz. bottle, 50 cents; 2 oz. bottle, 25 cents·

In view of the above it would seem that the present is an opportune time to thoroughly test this preparation, which has excited so much favorable comment in recent years. It is reported to be unusually potent and free from danger both of habit-forming and immediate evil effect. Neurosine possesses, if this is true, a wide field of usefulness in the treatment of nervous and mental cases. Now, that its use means an economy of from 25 per cent. to 200 per cent., is an excellent time to become acquainted with its merits.

### TWO STRENGTHS OF PITUITRIN.

As is well known to medical practitioners, Parke, Davis & Co. have for several years manufactured a standard pituitary extract under the name of "Pituitrin." The product is prepared from the posterior lobe of the pituitary gland and has come into extensive use in the treatment of delayed parturition or uterine inertia· Being specifically intended for use in obstetrical work, this preparation will hereafter be designated, in label and literature, as Pituitrin "O" (Pituitrin obstetrical).

Announcement is now made of a second preparation of the pituitary gland, to be known as Pituitrin "S" (Pituitrin surgical). This product is approximately twice the strength of the former and is indicated specifically in the treatment of post-operative intestinal paresis, vesical atony, hemorrhage shock. Because of its exceptional potency it should not be used in obstetrical practice. In order that it may be readily distinguished from Pituitrin "O" (obstetrical) the carton labels are printed with red letters on white paper.

Both Pituitrin "O" and Pituitrin "S" are physiologically tested for activity.

Pituitrin "O" is supplied in ampoules of 1 mil (1 Cc.) and 1-2 mil (1-2 Cc.), respectively, and in bottles of 1-2 ounce. Pituitrin "S" is supplied in ampoules of 1 mil (1 Cc.) only.

---

IN PRURITUS—Even is severe forms of genital, anal, diabetic, eczematous itching, K-Y Lubricating Jelly in a great majority of cases, will bring relief, or at least grateful alleviation.

To anoint the skin in these conditions, K-Y Lubricating Jelly is not only effective, but convenient and economical, since it can be used without staining or soiling the bed clothes or the patient's linen. If the part is washed before each application, the best results are obtained.

### CHRONIC CONSTIPATION OF INFANTS—ITS PROPHYLAXIS.

Many an infant is constipated, but just naturally "outgrows it."

This is usually the case where the causative factor is merely the overcrowding of the colon in a small pelvis, for the size of the colon develops as time goes by, more slowly than does the rest of the body.

On the other hand, many a chronically constipated infant grows up in his ways into an intractably constipated adult, so that anatomic structure is not the only consideration.

Prophylaxis, therefore, is the thing. Some physicians get the mother to hold the infant over its chamber morning and night immediately after feeding, long before it has mastered the secret of bowel control.

As a measure of prophylactic training in this connection, there is nothing which will help more than INTEROL. For without cathartic action, it lubricates the fecal mass, soft and plastic, into the sigmoid and rectum, whence its expulsion is a comparatively easy matter, in the absence of congenital defects.

This measure, in conjunction with proper feeding or diet and general hygiene, will help the infant or young child to establish the habit of regular stool so valuable to him in later life.

---

### AN ALTERATIVE OF VALUE.

A generation of physician's has tested with complete satisfaction the alterative powers of IODIA (Battle), and the result is that today it is more popular with exacting clinicians than ever before.

IODIA (Battle) exerts a definite influence in late syphilis, when iodine is urgently needed, and in other conditions requiring agents of an alterative character. Crowd it in syphilitic deposits.

